# Editorial: Machine Learning and Mathematical Models for Single-Cell Data Analysis

**DOI:** 10.3389/fgene.2022.911999

**Published:** 2022-06-03

**Authors:** Le Ou-Yang, Xiao-Fei Zhang, Jiajun Zhang, Jin Chen, Min Wu

**Affiliations:** ^1^ Guangdong Key Laboratory of Intelligent Information Processing, Shenzhen Key Laboratory of Media Security, Guangdong Laboratory of Artificial Intelligence and Digital Economy(SZ), College of Electronics and Information Engineering, Shenzhen University, Shenzhen, China; ^2^ School of Mathematics and Statistics and Hubei Key Laboratory of Mathematical Sciences, Central China Normal University, Wuhan, China; ^3^ Guangdong Province Key Laboratory of Computational Science, School of Mathematics, Sun Yat-sen University, Guangzhou, China; ^4^ Institute for Biomedical Informatics, University of Kentucky, Lexington, KY, United States; ^5^ Institute for Infocomm Research (I2R), A*STAR, Singapore, Singapore

**Keywords:** single-cell omics data, machine learning, mathematical modelling, data integration, network modeling

Understanding how individual cells communicate with each other and respond to evolution and perturbations is a central challenge of biology (Altschuler and Wu, 2010). Due to the heterogeneity of cells, studying a bulk population of cells may confound the variability of cell-type compositions, single cell analysis has the potential to enable a more systematic study of the inner workings of biological systems, and allows us to uncover the underlying mechanisms for cellular functions and biological processes such as cell differentiation and disease development. In the past decade, advances in single-cell isolation and sequencing technologies have enabled the assay of DNA, mRNA, and protein abundances at single-cell resolution, which promote the study of genomics, transcriptomics, proteomics and metabolomics at the sinlge cell level. For example, single-cell genomic analysis can shed light to the genomic variability of individual cells, while single-cell transcriptomic and proteomic analysis can help to reveal the types and functional states of individual cells (Shapiro et al., 2013). However, processing single-cell data of high dimensionality and scale is inherently difficult, especially considering the degree of noise, sparsity, batch effects and heterogeneity in the data (Amodio et al., 2019). Thus, there is an urgent need for developing computational models which can handle the size, dimensionality, and various characteristics of single-cell data. In this Research Topic of Frontiers in Genetics on “*Machine Learning and Mathematical Models for Single-Cell Data Analysis*,” we have collected eight manuscripts that used machine learning algorithms or mathematical models to solve problems in single cell analysis.

Single-cell and whole tissue RNA sequencing technologies enable the Research Topic of detailed information about biological processes at genomic and transcriptomic levels. Besides, existing microscopy and cell-resolution imaging techniques allow the high-quality characterization of morphology and physiology at the level of extended fragments of tissues and organs. Bobrovskikh et al. summarized the potential of single-cell technologies together with advanced imaging techniques for computational modelling in plants. They reviewed currently available single-cell data analysis approaches, advanced imaging technologies in plant research with single-cell resolution and cell-based modelling approaches. They shown how the combination of single-cell data, morphometric data and cell-based models help to expand the understanding of tissue and organ morphogenesis.

Tissues are constituted of heterogeneous cell types. Although single-cell RNA sequencing has paved the way to a deeper understanding of organismal cellular composition, the high cost and technical noise have prevented its wide application. As an alternative, computational deconvolution of bulk tissues can be a cost-effective solution (Jin and Liu, 2021). Liu et al. proposed a deconvolution method, named DecOT, to characterize the cell type composition from bulk tissue RNA-seq data. DecOT uses the optimal transport distance as a loss and applies an ensemble framework to integrate reference information from scRNA-seq data of multiple individuals. Experiment results on real data sets demonstrated that DecOT outperformed other existing methods and was robust to the choice of references.

The development of single-cell sequencing technologies promotes the researches on developmental physiology and disease (Potter, 2018), but the spatial information of individual cells is lost due to the tissue dissociation processes in these technologies. Highly multiplexed imaging technologies, such as imaging mass cytometry (IMC), are powerful tools to exploit the composition and interactions of cells in tumor microenvironments at subcellular resolution. However, due to the high resolution and large number of channels, how to process and interpret IMC image data still remains challenging (Chang et al., 2017). To improve the accuracy of single cell segmentation, which is a critical step to process IMC image data, Xiao et al. developed a deep neural network (DNN)-based cell segmentation method, named Dice-XMBD. Dice-XMBD is marker agnostic and can perform accurate cell segmentation of IMC images of different channel configurations without modification.

Advances in single-cell RNA-sequencing (scRNA-seq) technology provided an unprecedented opportunity for researchers to study the identity and mechanisms of single cells (Morris, 2019). Besides scRNA-seq data, spatial location data can also provide important information on the cells’ micro-environment and cell-cell interactions (Mayr et al., 2019), which can contribute to cell type identification. Oh et al. proposed a hybrid clustering approach, named single-cell Hybrid Nonnegative Matrix Factorization (scHybridNMF), to perform cell clustering by jointly processing cell location and gene expression data. ScHybridNMF combines sparse nonnegative matrix factorization (sparse NMF) with k-means clustering to cluster high-dimensional gene expression and low-dimensional location data. Experiment results on simulated and real data sets demonstrate the effectiveness of scHybridNMF in detecting cell clusters.

The communication between cells plays a vital role in the development, physicology, and pathology of muticellular organisms. Single-cell RNA-sequencing (scRNA-seq), which measures the expression levels of a great number of genes across various cell types at single-cell resolution, provides a great opportunity to study the cell-cell communication between interacting cells and the signaling response governed by intracellular gene regulatory networks (GRNs) (Shao et al., 2020). Identification the changes of intercellular signaling across different conditions is crucial for understanding how distinct cell states respond to evolution, perturbations, and diseases. Wang et al. generalized their previously developed tool CellChat to enable a flexible comparison analysis of cell-cell communication networks across multiple conditions, which facilitated the detection of signaling changes of cell-cell communication in response to biological perturbations. By studying the signaling changes across three mouse embryonic developmental stages, four time points after mouse spinal cord injury, and patients with different COVID-19 severities (i.e., control, moderate, and critical cases), they verified the effectiveness of their proposed approaches. To infer the changes of GRNs between two different states, Liu et al. proposed a general differential network inference framework, named weighted joint sparse penalized D-trace model (WJSDM). WJSDM can directly infer the differential network between two different states by integrating multi-platform gene expression data and various existing biological knowledge. By applying WJSDM to the gene expression data of ovarian cancer and the scRNA-seq data of circulating tumor cells of prostate cancer, and infer the differential network associated with platinum resistance of ovarian cancer and anti-androgen resistance of prostate cancer, the authors found some important biological insights about the mechanisms underlying platinum resistance of ovarian cancer and anti-androgen resistance of prostate cancer.

Recent advances in experimental biology have generated huge amounts of data. For example, Microwell-Seq, a single-cell RNA-sequencing technology, has been used to analyze the transcriptome of more than 400,000 mouse single cells, covering all major mouse organs (Han et al., 2018). There is an urgent need for next generation methods to deal with large, heterogeneous and complex data sets Camacho et al. (2018). As a promising data processing method, deep learning methods have been employed in biological data processing (Eraslan et al., 2019). However, the deep learning methods usually run as a “black box,” which is hard to interpret. The capsule network (CapsNet) is a newly developed deep learning model for digital recognition tasks (Sabour et al., 2017). Wang et al. (2020) proposed a modified CapsNet model, called single cell capsule network (scCapsNet), which is a highly interpretable cell type classifier, with the capability of revealing cell type associated genes by model internal parameters. Based on CapsNet and scCapsNet, Wang et al. proposed a deep learning classifier and data integrator, named MultiCapsNet. The MultiCapsNet model could integrate multiple input sources and standardize the inputs, then use the standardized information for classification through capsule network. The experiment results on three data sets with different data type and application scenarios proved the validity and interpretability of MultiCapsNet.

Cancer immunotherapy has shown to elicit substantial response to many cancers and has led to significant increases in quality of life for cancer patients. This is especially true of checkpoint therapy, which causes tumor regression in previously untreatable cancers. However, the potential mechanisms of checkpoint therapy are still being investigated and there are as of yet few prognostic markers for response (Bai et al., 2020). Immune checkpoint therapies such as PD-1 blockade have vastly improved the treatment of numerous cancers, including basal cell carcinoma (BCC). However, patients afflicted with pancreatic ductal carcinoma (PDAC), one of the deadliest malignancies, overwhelmingly exhibit negative responses to checkpoint therapy. Liu et al. sought to combine data analysis and machine learning to differentiate the putative mechanisms of BCC and PDAC non-response. By comparing two recent single-cell transcriptomic datasets of PDAC and BCC, the authors identified some potential biomarkers and mechanisms related to BCC and PDAC non-response. By utilizing machine learning classification algorithms, they also discovered that PDAC displays greater similarities to melanoma, which is highly immunogenic and undergoes rapid metastasis, than to BCC (Dollinger et al., 2020).

In summary, this Research Topic covers various aspects of machine learning models, including supervised and unsupervised approaches and their applications for single-cell data analysis, which paves the way for using machine learning and mathematical models in service of various tasks towards single cell analysis. We hope the readers from bioinformatics and the domain specific researchers will be benefitted by reading articles included in this Research Topic.
